# Association between COVID-related health literacy and vaccine acceptance: results of a nationwide cross-sectional study in Hungary

**DOI:** 10.1186/s12889-026-26491-5

**Published:** 2026-02-07

**Authors:** Yusuf Elisha Karu, Gabriella Mátyás, Ferenc Vincze, Róza Ádány, Éva Bíró

**Affiliations:** 1 Department of Public Health and Epidemiology, Faculty of Medicine, University of Debrecen, Debrecen, Hungary; 2 International and Private Care, Great Ormond Street Hospital Charity, London, UK; 3 Doctoral School of Health Sciences, University of Debrecen, Debrecen, Hungary; 4 HUN-REN-UD Public Health Research Group, Department of Public Health and Epidemiology, Faculty of Medicine, University of Debrecen, Debrecen, Hungary; 5 National Laboratory for Health Security, Center for Epidemiology and Surveillance, Semmelweis University, Budapest, Hungary; 6 Department of Preventive Medicine and Public Health, Semmelweis University, Budapest, Hungary

**Keywords:** COVID-related health literacy, Vaccine acceptance, Vaccine hesitancy, Vaccination, HLS-COVID-Q22

## Abstract

**Background:**

Health literacy is essential for understanding the coronavirus disease 2019 (COVID-19), dealing with health information, and making vaccine-related decisions. We aimed to determine the level of COVID-related health literacy, identify its determinants, and investigate the association between COVID-related health literacy and vaccine acceptance from a sample of adults in the Hungarian population.

**Methods:**

In 2022, we surveyed 1200 Hungarian adults aged 18 and older. It was a probability sample of the Hungarian adult population. A polling company conducted computer-assisted personal interviews to collect data. The questionnaire included items on socio-demographics, health status, vaccine acceptance and COVID-related health literacy. The determinants of COVID-related health literacy and its association with vaccine acceptance were investigated by binary logistic regression.

**Results:**

Almost half of the sample (43.6%) had a sufficient level of COVID-related health literacy. Being female (odds ratio, OR 1.46), having a tertiary education (OR 2.26), belonging to the normal social status in society (OR 1.74), average or (very) good subjective perception of family wealth (OR 2.08 and 3.28, respectively) and (very) good self-perceived health status (OR 2.58) were associated with a sufficient level of COVID-related health literacy. Vaccine acceptance was positively associated with a problematic or sufficient level of COVID-related health literacy (OR 2.55 and 2.57, respectively), older age (OR 1.02) and completing tertiary education (OR 1.98), while living in smaller cities (OR 0.52) or villages (OR 0.44) and being unemployed (OR 0.47) were negatively associated with vaccine acceptance.

**Conclusions:**

Vaccine acceptance was positively associated with COVID-related health literacy and negatively associated with unemployment and living in a small town or a village. Therefore, we advocate for programs that targeting these groups to enhance vaccine acceptance.

## Background

On March 11, 2020, the World Health Organization (WHO) declared the coronavirus disease 2019 (COVID-19) a pandemic, expressing deep concern over its rapid spread and severity, as well as the alarming levels of inaction [1]. The outbreak sparked a global crisis, profoundly disrupting many aspects of daily life. Vaccination has since proven to be a highly effective measure in preventing severe illness and reducing COVID-19-related mortality [2–4]. Although vaccines provide effective protection, a global survey found moderate vaccine acceptance, with rising hesitancy in many countries [5].

Vaccination programs could be successful only when vaccination acceptance and coverage are at high rates, for this reason, the WHO listed vaccine hesitancy as one of the 10 threats to global health in 2019 [6]. Vaccine hesitancy refers to a reluctance or delay in vaccination despite its availability. The problem is multifaceted and influenced by geographical, time- and vaccine-specific factors, making it complex and context-dependent [7]. Vaccine acceptance is “the degree which individuals accept, question, or refuse vaccination” [8, 9].

Vaccine acceptance and hesitancy can be affected by the following factors: socioeconomic; cultural and demographic (age, sex, ethnicity) elements; religious beliefs; perceived risk and severity; trust in authorities; accessibility and cost of vaccines; and exposure to information and misinformation [10–13]. The vaccine acceptance rate refers to the percentage of people who are willing to get vaccinated when the vaccine is offered [9]. It is important to note that vaccine acceptance rate does not equal vaccine rate, which denotes the percentage of a population that has received a specific vaccine. Vaccine uptake disregards the choice to accept or decline a vaccination, and some individuals get vaccinated despite their doubts and concerns. Therefore, vaccine rates cannot reflect the level of vaccine hesitancy or vaccine acceptance [9].

The global vaccine hesitancy for COVID-19 vaccines was 25% between 2020 and 2021, based on results from studies conducted among the general population, students, and healthcare workers [14]. Within Europe, vaccine hesitancy ranged from 6.4% (Spain) to 61.8% (Bulgaria) [15]. The COVID-19 vaccine acceptance rate varied widely around the world from 13 to 21% (Iraq, Cameroon, Bahrain, Algeria, Lebanon and Senegal) to 91–97% (Canada, Ethiopia, Tunisia, Niger, Nepal and Vietnam) [16]. Recent publication found that globally, the COVID-19 vaccine acceptance rate was the highest among healthcare professionals, 71.4%; followed by students with 64.7% [17]. The Hungarian and French acceptance rate of the COVID-19 vaccination was particularly low (47%) based on a survey in eight countries, the highest acceptance rate was measured in Denmark (83%) [18]. In a Hungarian research conducted in 2021 among the general population, 48.2% of individuals reported a willingness to receive the COVID-19 vaccination, individuals over 59 years and those with higher education showed higher desire to receive the vaccine [19]. Based on another Hungarian study, government supporters, individuals aged 60 and above, and COVID-19 survivors demonstrated a higher likelihood to accept vaccination [20]. The COVID-19 vaccine acceptance rate was higher among Hungarian medical students (88.6%) than among the general population – just as seen above in the global trend – according to a survey in the first semester of the 2021–2022 academic year [21].

Healthcare professionals serve as a reliable source of information regarding vaccines; they play an essential role in promoting vaccine acceptance among the general population. The significance of healthcare professionals’ communication skills and their understanding of vaccines often remains undervalued [22]. The COVID-19 infodemic – which refers to the excessive amount of information, including false or misleading content, present in both digital and physical contexts during a disease outbreak – puts in the light the importance of adequate health communication and, in certain cases, the low level of health literacy (HL) among the general population [23].

HL, which, according to Sørensen et al., “is linked to literacy and entails people’s knowledge, motivation, and competence to access, understand, appraise, and apply health information in order to make judgments and take decisions in everyday life concerning healthcare, disease prevention, and health promotion to maintain or improve quality of life during the life course” [24] (p. 3), has an effect on vaccine hesitancy or acceptance [25]. Recent investigations highlighted COVID-19 vaccine acceptance significantly associated with both general HL and COVID-19 vaccine HL [16]. Although the two HLs are different, both are relevant in pandemic times: general HL is a broader concept that helps people make health-related decisions, and COVID-19 vaccine HL is a specialised HL focusing on COVID-19 vaccination knowledge, attitudes, and behaviours; they both play an important role in vaccine-related decisions [24, 26]. General HL extends beyond vaccinations and also influences other health-related decisions and behaviours. Individuals with a sufficient level of HL can make suitable health-related decisions and improving HL can increase the COVID-19 vaccination’s acceptance [16]. HL can also act as a social vaccine to complement biomedical vaccines to empower individuals and help them to deal with health information regarding COVID-19. Besides protecting themselves, health literate individuals can also protect the community’s health, including that of high-risk and vulnerable groups [27].

Despite the clear link between HL and acceptance of vaccinations, the level of HL is far from satisfactory. According to the results of the Health Literacy Survey 2019–2021 (HLS_19_), more than half of the Hungarian adult population (59%) had an “excellent” and “sufficient” level of general HL; meanwhile 30% of them had “problematic” and 11% of them had an “inadequate” level of HL [28]. This distribution was very similar to the international average, where 55% belonged to the excellent and sufficient, 33% to the problematic and 13% to the inadequate category [29].

In light of the vital role that HL plays in the COVID-19 pandemic, it is important to measure the COVID-related health literacy (COVID-HL) and develop and validate pandemic-related HL tools as well [30]. The concept of general HL is too broad for pandemic situations; however, COVID-HL specifically evaluates decision-making during the pandemic. This specialised HL is essential for effective pandemic control because it assesses the competencies necessary to prevent infection, reduce transmission, and increase vaccine acceptance [31, 32]. In various studies, questionnaires used to assess the level of COVID-HL were based on the HLS-EU-Q47 questionnaire from the European Health Literacy Survey, which measures HL in general population [31, 33–37]. Some studies used only the questionnaire coined by the research team [38]. Some other studies only investigated knowledge about COVID-19 [39] or looked at the relationship between HL and knowledge or attitudes about the coronavirus [40–44]. In some studies, only digital dimension of COVID-HL was measured [45, 46].

Seeing the importance of HL, especially COVID-HL, and the low level of COVID-19 vaccine acceptance, the aim of our study was to determine the level of COVID-HL in Hungary, identify its determinants and investigate the association between COVID-HL and vaccine acceptance from a representative sample of adults in the Hungarian population.

## Methods

### Study design and sampling

Data were collected using computer-assisted personal interviews (CAPI) conducted by the TÁRKI Social Research Institute, a professional polling company; a total of 1200 Hungarian adults (response rate calculated based on the ratio of the checked and approved interviews to the number of people contacted: 53%) participated in the survey [47]. The data collection took place between January 28, 2022, and February 16, 2022. The sample was drawn using a probability sampling method to ensure representativeness of the Hungarian population aged 18 years and older with permanent residence in non-institutional settings. The sampling was based on the most recent population census data from the Hungarian Central Statistical Office, reflecting the national distribution of sex, age groups, regions, and settlement types. A proportionally stratified probability sampling method was used. The stratification process began with the selection of settlements, ensuring representation of the various types of settlements in Hungary. Within each region, the number of individuals to be sampled from each type of settlement was determined based on population proportions. The guiding principle for determining the number of respondents was to ensure that the distribution of participants from each region and by type of municipality within each region mirrored the distribution of the target population. In the last step, weights were applied to ensure the estimates reflected the general adult Hungarian population in terms of age, sex, education level, and settlement type. The Medical Research Council Scientific and Research Committee, Hungary, approved the study (IV/184-1/2022/EKU). Informed consent was obtained from all participants in accordance with the Declaration of Helsinki.

### Domains of the questionnaire

#### Sociodemographic and self-perceived health related data

Regarding sociodemographic data, the following topics were covered: age, sex (male, female), education level (primary, secondary or tertiary), having children (yes, no), previous training in a healthcare profession (yes, no), type of settlement (capital, city with county rights, city and village), employment status (active, unemployed, retired, student), number of family members living together and partnership/relationship (single living alone, single living in a shared household, living with a legal partner in a shared household, with legal partner but not living together). Self-perceived level in society was measured on a 1–10 scale, where 1 represents the lowest, while 10 is the highest level in the society. A score below 5 indicates “low” social status, according to the HLS_19_ methodology for identifying potentially vulnerable groups. A score equal or above 5 is assessed as “normal” social status [29]. Subjective perception of family wealth was assessed on a scale of 1 to 5, where 1 indicated “very good” and 5 indicated “very bad” [48]. Self-perceived health – as in the case of the European Health Interview Survey 2019 – was measured on a 5-point Likert-scale, from “very bad” to “very good” [48]. 

#### Sources of information, coronavirus infection and risk perception

We used multiple choice questions (MCQs), where the number of answers was not limited, to ask participants whether they had consulted a specified list of sources of information [29] on COVID-19. The list consists of (1) health professionals, (2) official website or person, (3) television or radio, (4) digital sources, (5) international sources, like WHO, (6) lay people, (7) written materials, (8) telephone information line, (9) complementary, alternative or unconventional practitioners, (10) celebrities or influencers, 11) does not seek any information from any sources. Using this list, respondents were also asked in a separate question with a maximum of three answers, which sources they considered most credible. Here the 12th option was “don’t trust any of them”.

Infection-related questions included whether they had ever contracted the virus causing COVID-19 and if they had been in contact with anyone who was infected. It was possible for them to respond through the following options: (i) confirmed with mild symptoms; (ii) confirmed with severe symptoms; (iii) had similar symptoms but was not tested; or (iv) probably not [49]. (All tests, whether conducted by healthcare professionals or self-administered, were classified as confirmed cases.)

Participant’s perception of whether they were at risk of the infection was assessed using an MCQ based on the following risk factors: (i) yes, because of my age; (ii) yes, because I have a chronic disease/am obese; (iii) yes, because of my job; (iv) no; and (v) I don’t know [49]. 

#### COVID-related health literacy

We used the Hungarian version of the HLS-COVID-Q22 questionnaire to assess COVID-HL in the general population [34, 47]. The questionnaire uses the comprehensive model of HL [24]. It contains 22 items, which are based on an adapted version of the HLS-EU-Q16 questionnaire for COVID-19, with six additional questions on COVID-19 (e.g. How easy or difficult it is for you to … find information about the coronavirus on the internet?; … understand information in the media on how to protect yourself against coronavirus infection?; … judge what protective measures you can apply to prevent a coronavirus infection?). The items are answered on a 4-point Likert scale (1 – “very difficult”, 4 – “very easy”). The mean of the scale responses measures the level of COVID-HL, where higher values indicating better COVID-HL. In the sample, a mean response of ≤ 2.5 denoted “inadequate”, a mean of > 2.5 – < 3 indicated “problematic”, and a mean of ≥ 3 signified “sufficient” COVID-HL, consistent with the original German study [34, 47]. The COVID-HL levels were defined using the categories of the HLS-EU-Q16 questionnaire (inadequate, problematic, sufficient and excellent), but the sufficient and excellent categories were merged to sufficient as Röthlin et al. suggested [50, 51]. For performing a binary logistic regression, these three groups were reclassified into two categories: inadequate-problematic and sufficient COVID-HL.

#### Vaccine acceptance

The respondents were also asked about their intentions regarding COVID-19 vaccinations (yes/no/have not decided yet) [52]. We also examined the respondents’ reasons for opting out of vaccinations (if they answered no or have not decided yet for the previous question). They could choose the relevant options from this list [53]: (1) I am worried about the later, as yet unknown, effects of the coronavirus vaccine. (2) I am afraid of the side effects of the coronavirus vaccine. (3) I don’t trust vaccines generally. (4) The risk of me developing a serious illness from the coronavirus is small. (5) The probability that I will catch the coronavirus is small. (6) They exaggerate the impact of the coronavirus. (7) I don’t think it would prevent me from catching the coronavirus. (8) Due to my condition, the vaccine is not safe for me. (9) Herd immunity protects me even if I don’t get vaccinated. (10) I am protected because I went through the infection. 11) I don’t trust the authorities that approve vaccinations. 12) It’s impossible/hard for me to go get the vaccine. 13) Other. Respondents were given the opportunity to select more than one response.

They were also asked as an MCQ about their motivations for vaccination, where the potential factors could be marked from the options below [53]: (1) So that I don’t get infected or become seriously ill. (2) So that I can return to my normal family and community life. (3) To protect others from infection. (4) Because the protection against the epidemic will not be effective until the majority is vaccinated. (5) Because there is an increased risk of transmitting the infection during my work. (6) Because I usually take the vaccinations recommended for me. (7) To be safe again when I leave my home. (8) So that my child’s studies are not affected by my infection. (9) To get back to work. (10) I wouldn’t get the vaccine. 11) Other.

Lastly, the circumstances in which they would choose to vaccinate (if they answered no or have not decided yet for getting the vaccine) were investigated with an MCQ containing the following answers [53]: (1) It would be convincingly proven that vaccination reduces the risk of coronavirus infection. (2) It would be convincingly proven that vaccination reduces the risk of serious illness. (3) They would convincingly prove that vaccination is safe. (4) If it were convincingly proven to me that there is little risk of side effects from vaccination. (5) The scientists would recommend that I vaccinate myself. (6) The health workers would recommend that I get vaccinated. (7) I would have easy access to the vaccine at home or at work. (8) I would need a protection certificate. (9) They would give me some kind of gift or compensation for it. (10) I could participate in a raffle. 11) It would be mandatory. 12) I wouldn’t get the vaccine in any case. 13) Other.

### Statistical analyses

The sociodemographic and health-related characteristics, COVID-19 infection, risk perception and sources of information, COVID-HL and vaccine acceptance were described using statistically weighted frequencies by age, sex, education level, and settlement type (%) with the corresponding 95% confidence intervals (95% CI). The determinants of COVID-HL and the relationship between COVID-HL and vaccine acceptance were investigated by multivariate binary logistic regression adjusted for age, sex, education, healthcare training, type of settlement, number of family members living together, partnerships, employment status, social status, having children, subjective family wealth, and self-perceived health. The assumptions of the logistic regression analysis were tested with linktest to detect specification errors and to perform a goodness-of-fit test, and collinearity diagnostics, all of which were met. The data analysis was performed using the Stata/IC 16 statistical software’s survey data analysis module (StataCorp LLC, Stata Statistical Software: Release 16. College Station, TX: USA).

## Results

### Descriptive statistics

The sample comprised 1200 adult participants from Hungary. The age ranged from 19 to 93 years, with an average age of 48.73 years. The majority of the sample were female. The sample showed that most of the individuals had secondary education. Individuals with healthcare training constituted only 12.7% of our sample and majority of respondents lived in urban areas. The average number of individuals living in a household was 2.18, with a maximum of 8 people recorded. Around one-third of the respondents did not have children. Over half of the participants resided with a legal or de facto partner in a shared household. The majority of participants were actively employed. About one-quarter of the participants belonged to the low social status category. More than half of the participants rated their family wealth as average. Almost two-third of the respondents assessed their health status as (very) good. The main characteristics of our sample are presented in Table 1.

**Table 1:** Main characteristics of the study sample (*N*=1200)

Variable		Unweighted percentage (95% CI)	Weighted percentage (95% CI)^*^
Sex	Male	39.3 (36.5–42.1)	46.6 (43.4–49.9)
	Female	60.8 (57.9–63.5)	53.4 (50.1–56.7)
Education level	Primary education	12.8 (11.1–14.9)	25.8 (22.5–29.5)
	Secondary education	70.9 (68.3–73.4)	56.5 (52.9–59.9)
	Tertiary education	16.3 (14.3–18.4)	17.7 (15.3–20.4)
Training in a health profession	Yes	15.2 (13.3–17.3)	12.7 (10.8–14.9)
	No	84.8 (82.7–86.7)	87.3 (85.1–89.3)
Partnership	Single living alone	40.5 (37.7–43.3)	40.4 (37.2–43.5)
	Single living in a shared household	3.8 (2.8–4.9)	4.1 (2.9–5.7)
	Living with a legal partner in a shared household	53.7 (50.9–56.5)	53.6 (50.4–56.8)
	With legal partner but not living together	2.1 (1.4–3.1)	1.9 (1.3–3.0)
Employment status	Active	67.3 (64.6–69.9)	67.2 (66.7–72.5)
	Unemployed	6.0 (4.8–7.5)	8.0 (6.1–10.4)
	Retired	24.7 (22.3–27.2)	22.3 (19.1–24.1)
	Student	2.0 (1.3–2.9)	2.5 (1.6–3.9)
Type of settlement	Capital	17.9 (15.9–20.2)	18.1 (15.6–21.0)
	City with county rights	18.5 (16.4–20.8)	19.2 (16.7–21.9)
	City	33.1 (30.5–35.8)	32.7 (29.7–35.7)
	Village	30.5 (27.9–33.2)	30.0 (27.2–32.9)
Having children	Yes	71.1 (68.5–73.6)	66.2 (62.9–69.3)
	No	28.9 (26.4–31.6)	33.8 (30.7–37.6)
Self-perceived health	(Very) bad	7.9 (6.5–9.6)	9.0 (7.1–11.3)
	Fair	32.0 (29.4–34.7)	29.1 (26.0–32.3)
	(Very) good	60.1 (57.3–62.8)	61.9 (58.5–65.3)
Social status	Low	24.2 (21.9–26.7)	28.5 (25.3–31.9)
	Normal	75.8 (73.3–78.1)	71.5 (68.1–74.7)
Subjective perception of wealth	(Very) good	28.3 (25.9–30.9)	27.9 (25.0–31.0)
	Average	56.0 (53.2–58.8)	54.0 (50.5-57.4)
	(Very) bad	15.7 (13.7–17.8)	18.1 (15.4–21.2)

95% *CI* 95% confidence interval

*Weighted by age, sex, education, and place of residence

### The COVID-related health literacy and its determinants

It was found that nearly half of the sample (43.6%, 95% CI: 40.4–46.8) had a sufficient, about a quarter (25.2%, 95% CI: 22.5–28.1) had inadequate and a third of them (31.3%, 95% CI: 28.3–34.3) demonstrated problematic COVID-HL (*n* = 1180). For the respondents, the easiest item from the HLS-COVID-Q22 questionnaire was finding information about coronavirus on the internet, and the most difficult task was judging if information on the coronavirus and the coronavirus epidemic in the media is reliable.

Only 5.9% (95% CI: 4.6–7.5) of our sample had a confirmed COVID-19 infection with severe symptoms, while an additional 26.3% (95% CI: 23.6–29.3) were confirmed cases with mild symptoms. Nearly half of the respondents, 45.3% (95% CI: 42.1–48.6), were aware of individuals having mild or no symptoms despite confirmed cases, while 33.1% (95% CI: 30.2–36.2) reported knowing individuals with severe symptoms.

Participants were asked to assess their perceived risk of COVID-19 infection. Of these 21.3% (95% CI: 18.9–23.9) identified their age as a risk factor, 17.1% (95% CI: 15.0–19.6) cited chronic disease or obesity as a risk factor, and 22.3% (95% CI: 19.7–25.1) attributed their risk to their occupation. Approximately 37.4% (95% CI: 34.3–40.6) did not perceive themselves as at risk for COVID-19 infection, whereas 11.4% (95% CI: 9.5–13.6) were uncertain about their risk status.

Analysis indicated that the odds of having sufficient COVID-HL were 1.46 times (95% CI: 1.11–1.90) larger for females than for males. Having a tertiary level of education (odds ratio, OR 2.26, 95% CI: 1.28–3.99) and a social status above the “low” level (OR 1.74, 95% CI: 1.20–2.51) significantly increased the likelihood of having sufficient COVID-HL. In comparison to individuals with (very) bad subjective perception of wealth, those with average or (very) good subjective perception of wealth demonstrated a higher likelihood of having higher literacy levels (OR 2.08, 95% CI 1.30–3.34; and 3.28, 95% CI 1.94–5.57, respectively). Those with (very) good health status has higher odds of having sufficient COVID-HL compared to those with (vey) bad health status (OR 2.58, 95% CI: 1.39–4.80). Table 2 provides more detailed illustrations.

**Table 2:** The determinants of sufficient COVID-related health literacy (ref. inadequate and problematic) (n=1164)

	OR	95% CI	*p*-value
Sex (ref. males)			
Female	1.46	1.11–1.90	0.006*
Age	1.00	0.99–1.01	0.966
Education (ref. primary)			
Secondary	1.46	0.90–2.35	0.124
Tertiary	2.26	1.28–3.99	0.005*
Professional healthcare training (ref. yes)			
No healthcare training	1.29	0.89–1.89	0.182
Type of settlement (ref. capital)			
City with county right	0.96	0.61–1.51	0.843
City	0.79	0.53–1.17	0.239
Village	1.16	0.76–1.75	0.491
Number of family members living together	0.92	0.77–1.09	0.340
Partnership (ref. living alone)			
Single living in shared household	0.52	0.25–1.08	0.080
Living with legal partner in a shared household	1.25	0.88–1.75	0.210
With legal partner but not living together	0.99	0.41–2.42	0.991
Having children (ref. having children)			
Do not have children	1.20	0.84–1.71	0.316
Employment status (ref. active)			
Unemployed	1.43	0.80–2.56	0.231
Retired	0.94	0.60–1.48	0.795
Student	2.38	0.86–6.61**	0.097
Social status (ref. low)			
Normal	1.74	1.20–2.51	0.003*
Subjective perception of wealth (ref. (very) bad)			
Average	2.08	1.30–3.34	0.002*
(Very) good	3.28	1.94–5.57	<0.001*
Have you searched for any information about COVID-19 from any source? (ref. yes)			
No	1.18	0.86–1.61	0.310
Have you had a coronavirus infection? (ref. confirmed mild/no symptoms)			
Confirmed with severe symptom	1.18	0.68–2.03	0.557
Had similar symptom but was not tested	0.97	0.65–1.46	0.890
Probably no	1.03	0.74–1.42	0.869
Do you know someone who has been infected by coronavirus? (ref. confirmed mild/no symptoms)			
Confirmed with severe symptom	1.11	0.83–1.50	0.484
Had similar symptom but was not tested	1.01	0.63–1.60	0.966
Probably no	0.85	0.55–1.31	0.452
Self-perceived health (ref. (very) bad)			
Fair	1.76	0.96–3.22	0.069
(Very) good	2.58	1.39–4.80	0.003*

*OR* odds ratio, 95% *CI* 95% confidence interval, *ref*. reference category

*p < 0.05, ** uncertainity is high due to the small number of people in the category

### COVID-19 vaccine acceptance and its determinants

Concerning the question about self-vaccination against COVID-19, 73.1% (95% CI: 70.1–75.9) of respondents expressed willingness to get vaccinated, nearly a quarter (23.5%, 95% CI: 20.8–26.4) did not want to get vaccinated, and a small proportion, 3.4% (95% CI: 2.4–4.8), have not decided about getting vaccinated.

The study examined which factors are associated with the probability of getting vaccinated against COVID-19: problematic COVID-HL (OR 2.55, 95% CI: 1.75–3.72), sufficient COVID-HL (OR 2.57, 95% CI: 1.79–3.69), older age (OR 1.02, 95% CI: 1.01–1.04), completing tertiary education (OR 1.98, 95% CI: 1.07–3.65). Predictors that decreased the likelihood of vaccine acceptance included an “unemployed” employment status (OR 0.47, 95% CI: 0.27–0.83), residing in a small city (OR 0.52, 95% CI: 0.32–0.83), or living in a village (OR 0.44, 95% CI: 0.27–0.72). The other included sociodemographic variables showed no association. Table 3 presents comprehensive information on the model.

**Table 3:** The determinants of COVID-19 vaccine acceptance (n=1173)

	OR	95% CI	*p*-value
COVID-HL (ref. inadequate)			
Problematic	2.55	1.75–3.72	<0.001*
Sufficient	2.57	1.79–3.70	<0.001*
Sex (ref. male)			
Female	0.78	0.57–1.06	0.106
Age	1.02	1.01–1.04	0.009*
Education (ref. primary)			
Secondary	1.29	0.81–2.05	0.293
Tertiary	1.98	1.08–3.65	0.029*
Professional healthcare training (ref. yes)			
No healthcare training	0.89	0.56–1.44	0.648
Type of settlement (ref. capital)			
City with county rights	0.66	0.38–1.14	0.137
City	0.52	0.32–0.83	0.007*
Village	0.44	0.27–0.72	0.001*
Number of family members living together	1.03	0.86–1.24	0.722
Partnership (ref. living alone)			
Single/living in shared household	1.00	0.49–2.06	0.991
Living with legal partner in a shared household	1.09	0.74–1.59	0.668
With legal partner but not living together	1.92	0.63–5.86^**^	0.251
Having children (ref. having children)			
Do not have children	0.77	0.52–1.14	0.188
Employment status (ref. active)			
Unemployed	0.47	0.27–0.83	0.009*
Retired	1.41	0.83–2.41	0.207
Student	1.67	0.61–4.56^**^	0.321
Social status (ref. low)			
Normal	1.42	0.96–2.09	0.079
Subjective perception of wealth (ref. (very) bad)			
Average	1.20	0.76–1.91	0.437
(Very) good	1.38	0.79–2.39	0.251
Self-perceived health (ref. (very) bad)			
Fair	1.54	0.85–2.80	0.157
(Very) good	0.86	0.47–1.59	0.629

95% *CI* 95% confidence interval, *OR* odds ratio, *COVID-HL* COVID-related health literacy, *ref*. reference category

* *p *< 0.05, ** uncertainty is high due to the small number of people in the category

### Further results: trusting in sources and reasons to get vaccinated or reject vaccination

We conducted some further investigations: we asked what the most searched and trusted sources of information were according to our respondents, and we wanted to know the probable reasons and circumstances that could influence the decision to get vaccinated against COVID-19. 

#### Searching for information about COVID-19

Over two-thirds (69.9%) of respondents said that they had searched for information about COVID-19. Health professionals were the most consulted (60.2%) sources. Nearly half of the participants tended to use television or radio (53.4%) and other digital sources (49.5%) besides health professionals when seeking information. Complementary, alternative, or unconventional practitioners, celebrities and telephone information line were the least utilised sources by participants. Table 4 presents an overview of information sources ranked by the number of participants who used them.

**Table 4:** Percentage of respondents who used the given information source*

Sources of information	Percentage (95% CI)
Health professionals	60.2 (57.0–63.3)
Television or radio	53.4 (50.2–56.6)
Digital sources	49.5 (46.2–52.7)
Official, government website/spokesperson	36.5 (33.5–39.6)
Lay people	29.4 (26.5–32.5)
International sources, like WHO	18.6 (16.2–21.3)
Written materials	10.8 (9.0–12.9)
Complementary, alternative or unconventional practitioners	9.5 (7.7–11.6)
Celebrities, influencers	4.6 (3.4–6.0)
Telephone information line	2.8 (1.9–4.1)
Refuse to answer	0.6 (0.3–1.3)

95% *CI* 95% confidence interval, *WHO * World Health Organization

*Weighted by age, sex, education, and place of residence

#### Trusting in sources

Health professionals were the most trusted sources (53.4%), followed by official, government websites, or spokepersons according to respondents. Only about a fifth of our participants considered TV, radio or other digital sources as reliable (20.6% and 16.0%, respectively). Table 5 presents a comprehensive overview of trusted information sources ranked by the number of participants who identified them as the most trustworthy.

**Table 5:** Percentage of respondents who considered the given information source the most trustworthy*

Sources of information	Percentage (95% CI)
Health professionals	53.4 (50.2–56.6)
Official, government website/spokesperson	28.4 (25.6–31.4)
Television or radio	20.6 (18.1–23.3)
Digital sources	16.0 (13.8–18.5)
International sources, like WHO	15.0 (12.9–17.4)
Lay people	7.4 (5.9–9.2)
Written materials	3.8 (2.8–5.3)
Telephone information line	3.2 (2.2–4.7)
Complementary, alternative or unconventional practitioners	3.1 (2.3–4.3)
Celebrities, influencers	1.3 (0.8–2.1)
Don’t trust any of the sources	9.7 (8.0–11.8)
Refuse to answer	0.9 (0.5–2.1)

95% *CI* 95% confidence interval, *WHO* World Health Organization

*Weighted by age, sex, education, and place of residence

#### Reasons of vaccine hesitancy

**Fig. 1:**
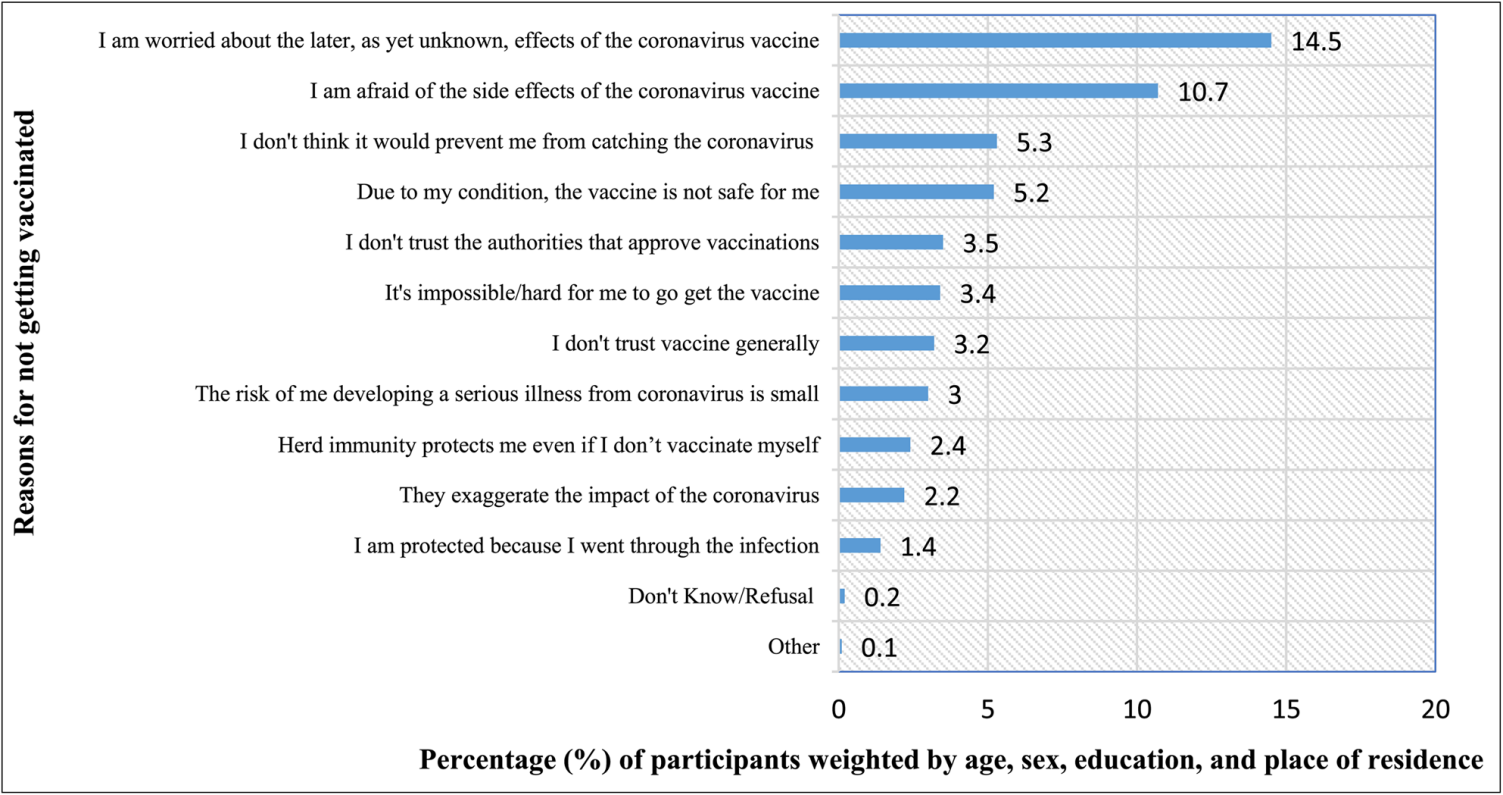
Percentage (%) of respondents based on their reasons for not getting vaccinated against COVID-19

We conducted further investigations into the probable reasons and circumstances that could influence the decision to get vaccinated against COVID-19. The most common reasons for COVID-19 vaccine hesitancy were “I am worried about the later, as yet unknown, effects of the coronavirus vaccine”, chosen by 14.5% (95% CI: 12.3–16.9) of participants, and “I am afraid of the side effects of the coronavirus vaccine” selected by 10.7% (95% CI: 8.8–13.0) of respondents. A comprehensive illustration is presented in Fig. 1.

#### Reasons for getting vaccinated against COVID-19

We also investigated why the participants had chosen to be vaccinated or why they intended to do so in the future. The most frequent reasons were as follows: 64.8% (95% CI: 61.6–67.8) said they would get vaccinated to avoid infection or serious illness, and 25.2% (95% CI: 22.6–28.0) said they would get vaccinated to protect others from infection. Further details can be found in Table 6.

**Table 6:** Percentage (%) of respondents who have chosen the given reason for getting vaccinated against COVID-19*

Why did you/would you get vaccinated against coronavirus?	Percentage (95% CI)
So that I don’t get infected or become seriously ill.	64.8 (61.6–67.8)
To protect others from infection	25.2 (22.6–28.0)
So that I can return to my normal family and community life	23.4 (20.8–26.2)
The protection against the epidemic will not be effective until the majority is vaccinated	22.2 (19.7–24.9)
There is an increased risk of transmitting the infection during my work	19.0 (16.6–21.6)
To be safe again when I leave my home	17.3 (15.1–19.9)
Because I usually take the vaccinations recommended for me	10.2 (8.5–12.2)
To get back to work	9.2 (7.5–11.2)
So that my child’s studies are not affected by my infection	3.3 (2.3–4.7)
I wouldn’t get the vaccine	16.8 (14.5–19.4)
Don’t know/refusal	0.7 (0.04–1.7)

*Weighted by age, sex, education, and place of residence, 95% CI 95% confidence interval

The participants were queried about the reasons under which they would opt for the COVID-19 vaccine. The following options were often selected: 12.6% (95% CI: 10.3–14.7) indicated they were not going to get the vaccine, but 6.4% (95% CI: 5–8.1) stated they would consider vaccination if it were convincingly demonstrated that the vaccine is safe, and 4.2% (95% CI: 3.1–5.6) would choose to be vaccinated if it were convincingly established that the risk of side effects is minimal. The remaining options were selected by less than 4% of the respondents and are therefore not discussed in detail.

## Discussion

Our study aimed to assess the level of COVID-HL in Hungary on a representative sample and identify its determinants. Our research also investigated the associations between COVID-HL and the willingness to be vaccinated against COVID-19.

Our findings indicate that fewer than half of our sample had sufficient COVID-HL, a proportion notably lower than the general HL level reported in the Hungarian HLS_19_ study, where 58.9% of participants demonstrated an adequate level of general HL [28]. As COVID-HL measures a specific set of skills related to the COVID-19 pandemic, not a broader set of skills as general HL does, this result could be expected. Our COVID-HL results seem to be in line with other similar international studies. Rosano et al. obtained a similar result to ours in Italy: 49.3% of their sample had an “excellent” level of COVID-HL [37]. Two studies, a Turkish and a German, measuring COVID-HL in the general population found a similar share of sufficient levels: 43.4% and 49.9%, respectively [34, 54]. De Gani et al. observed that the level of sufficient COVID-HL rose from 54.6% to 63.3% within the general Swiss German population from spring to winter 2020 [31]. A study in Indonesia among older people living in rural areas found that only 18% of the sample had a sufficient level of COVID-HL [36]. The results of studies in educational settings in Turkey and Hong Kong varied widely: 77.6% and 46.3% of their participants showed a sufficient level of COVID-HL, respectively [55, 56].

We found that certain sociodemographic characteristics were more likely to be associated with COVID-HL, such as being female, having obtained a tertiary education, having a social status above the “low” level and better subjective perception of wealth, which was associated with a sufficient level of COVID-HL. A German study had similar results, in their research mean scores for COVID-HL were significantly higher in females compared to males. Additionally, individuals with higher education levels demonstrated higher mean scores than those with lower levels of education, and those with a high subjective social status scored higher than those with a low subjective social status [57]. A Turkish study also found a relationship between age, education level, and COVID-HL [54]. An Italian study showed an association between age, financial deprivation and COVID-HL; sex and education level had a little impact [37]. A previous German study – just as another study in Spain – did not identify any association between COVID-HL and sex or age [32, 34]. Meanwhile, both studies [32, 34] found a relationship between education level – which is an associated variable with HL [33, 58, 59] – and COVID-HL. Our findings could be explained by the social gradient of HL – in this case, COVID-HL – which means that systematic differences in levels of HL across different social groups typically follow a gradient based on education, employment, or social status [29, 33].

Nearly three-quarters of our participants indicated their willingness to receive the COVID-19 vaccination. This result is in line with the findings of Bíró-Nagy et al. [20]. However, another Hungarian and an international study found only a 48.2% and 47% acceptance rate among their samples in Hungary [18, 19]. The difference in the results of the surveys may be explained by the time lag between the studies; that would mean an increasing acceptance rate of the COVID-19 vaccinations within the Hungarian population. This would be in line with the result of a recent meta-analysis, where the willingness to accept vaccination was 79.1% based on data from 23 countries, and they also found an increasing trend in vaccine acceptance [5]. This increasing trend in the COVID-19 vaccine acceptance rate among the Hungarian population may result from an increasing level of COVID-HL during the pandemic, similar to what was observed in Switzerland [31]. We can partially attribute this phenomenon to the ongoing media coverage of the pandemic and to the preventive measures.

Participation in healthcare training was not associated with COVID-HL or vaccine acceptance, which may suggest that the Hungarian curriculum is unable to enhance COVID-HL and vaccine acceptance enough.

Also, in our sample a positive correlation was identified between sufficient COVID-HL and willingness to be vaccinated against COVID-19. In addition to COVID-HL, various factors could affect vaccination decisions, including the type of vaccine available, political affiliation, and age group [30]. Some studies have also linked fear of injection to vaccine hesitancy [60]. It appears that the possible side effects and fear of unknown effects of the coronavirus vaccine influenced most of our respondents’ decision not to be vaccinated against COVID-19. These were also identified as factors contributing to COVID-19 vaccine hesitancy in a recent systematic review [11]. The most recurring reason for choosing to be vaccinated against COVID-19 was the fear of getting ill and preventing others from getting infected. It appears that reassuring individuals about vaccine safety and minimal side effects would also enhance vaccine acceptance in the case of coronavirus vaccine, as misinformation and fear of side effects contribute to vaccine hesitancy [11]. It emphasises the need to enhance health communication regarding vaccines and vaccination. Clear and inclusive health communication about vaccines using reliable and trusted information could lower vaccine hesitancy, making scientific information accessible for everyone, especially for vulnerable groups [11].

We identified multiple associations between certain sociodemographic characteristics and vaccine acceptance. The analysis indicated that individuals with an older age and tertiary level of education were more likely to be vaccinated against COVID-19. A systematic review and meta-analysis found positive association between higher education level and COVID-19 vaccine acceptance, while another systematic review found the opposite [11, 61]. Since behavioural factors such as risk perception, trust in authorities, and religion also play a role in vaccine acceptance or hesitancy beyond the level of education, the role of the latter may not be clear in systematic reviews. We can suppose that a higher education level is associated with better HL skills, but the vaccine decision-making process is so complex that a higher education level and understanding health-related information alone do not necessarily result in vaccine acceptance [22]. Older age – similarly to our results – was also linked to vaccine acceptance based on two systematic reviews [11, 61].

We observed negative associations with vaccine acceptance, particularly concerning unemployment status and residence in either smaller cities or rural areas. A study in the United Kingdom found that individuals with lower incomes and education levels demonstrated higher hesitancy and unwillingness to be vaccinated, respectively [49]. Unemployed people might feel less responsible towards their peers when they have less social contact, it could explain their vaccine hesitancy, but we have not examined this factor in our study. People living in smaller cities and rural areas have limited access to healthcare services, which reduces their chances of contacting healthcare professionals and receiving adequate information about vaccination, which can also result in vaccine hesitancy.

In our study the most trusted and consulted sources of information were health professionals. Nearly half of our respondents consulted television, radio, or other digital sources, but these were less trusted than health professionals; only a fifth of the respondents deemed them reliable. These results are in line with our previous findings from the Hungarian HLS_19_ study [28]. A considerable proportion of the respondents had never searched for information about COVID-19, but given the high level of media coverage of the topic, it was possible to obtain regular updates about the pandemic without actively searching for information. Nearly one tenth of our respondents did not trust any source of information, so it seems important to demonstrate that the information provided by official health-related campaigns and information sources is reliable and can help people make informed decisions regarding vaccinations. It is crucial to foster and sustain trust in the information channels utilised by official public institutions.

The difficulties in judging information as reliable underline the importance of health communication, especially about vaccines. Vaccine related information should be tailored to the general and specialised health literacy levels of individuals, particularly those with low levels. It is also important to use their preferred communication channels and targeted content. Since healthcare professionals are a reliable and demanded source of health-related information, their education in vaccinology and communication is essential [22]. The curriculum therefore should encompass how to adapt healthcare professionals’ communication for individuals with low health literacy [22]. Individuals having a lower level of HL may find it easier to make vaccine-related decisions after consulting simple health messages, as they struggle to interpret and evaluate health information. Improving the general population’s HL can ease vaccine-related decisions and result in an increased vaccine acceptance rate.

### Strengths and limitations

One of the strengths of our study lies in its implementation on a large, representative sample of the Hungarian population. The use of a questionnaire validated in multiple languages allows for comparability with other research findings. However, this study did not examine the relationship between general HL and COVID-HL, which could be valuable for understanding the overall influence of HL on vaccine acceptance or hesitancy. Furthermore, as it was a cross-sectional study, the causal relationship between the investigated variables cannot be proven. To prove the causality of the observed associations, further research is needed. Even though we used stratified sampling method and weighting procedure in our study to represent the Hungarian adult population, it cannot exclude selection bias attributable to non-responses, which should be considered when interpreting the findings. Although only a small proportion of respondents (12.7%) reported participation in healthcare training, this variable was included in the multivariate model as a potential confounder. Based on literature data, individuals with healthcare training may have higher baseline HL and different information-seeking patterns, which could influence the observed associations [21]. Nevertheless, the low prevalence of healthcare-trained respondents may limit the precision of estimates and the generalizability of this finding. Hence, we used self-reported questions; we cannot exclude self-reporting bias – when the information provided by the respondents does not totally reflect their behaviours or attitudes – from our findings. Social desirability may also have influenced the respondents since we collected data via personal interviews, and it could lead to overestimation of the vaccine acceptance rate. We also cannot exclude that some respondents misinterpreted some questions and intended differently. Our results can only be interpreted with the above limitations in mind.

## Conclusions

We recommend the implementation of strategic interventions to enhance COVID-HL and focusing more on individuals residing in small towns and villages, as well as the unemployed during vaccination promotion campaigns. Providing tailored information about vaccines to those with a low level of HL could enhance vaccination acceptance and lead to higher vaccine uptake. In the specific case of COVID-19, promoting COVID-HL could also increase COVID-19 vaccination rates.

In light of our findings, we also recommend further research focused on groups demonstrating vaccine hesitancy and inadequate or problematic levels of COVID-HL. This research should investigate their concerns and identify factors contributing to vaccine hesitancy within these populations.

## Data Availability

The datasets used and/or analysed during the current study are available from the corresponding author on reasonable request.

## Abbreviations

CAPI: Computer-assisted personal interview
CI: Confidence interval
COVID-HL: Coronavirus disease 2019 health literacy
COVID-19: Coronavirus disease 2019
HL: Health literacy
HLS_19_: Health Literacy Population Survey 2019–2021
HLS-COVID-Q22: 22-item questionnaire measuring the capacity to understand and utilize COVID-19-related health information in the general population based on the HLS-EU-Q16 questionnaire
HLS-EU-Q16: 16-item short version of the HLS-EU-Q47 questionnaire measuring health literacy in general populations from the European Health Literacy Survey
HLS-EU-Q47: 47-item questionnaire measuring health literacy in general populations from the European Health Literacy Survey
MCQ: Multiple-choice question
OR: Odds ratio
Ref.: Reference category
WHO: World Health Organization
